# How exposure to patient narratives affects stereotyped choices of primary care clinicians

**DOI:** 10.1371/journal.pone.0295243

**Published:** 2023-12-07

**Authors:** Deepon Bhaumik, Mark J. Schlesinger

**Affiliations:** Department of Health Policy and Management, Yale University, New Haven, Connecticut, United States of America; Lamar University, UNITED STATES

## Abstract

In this paper, we examine whether patient narratives alter the impact of stereotyping on choice of primary care clinicians: in this case, the common presumption that female doctors will be more attentive to empathic relationships with patients. 1052 individuals were selected from a nationally representative Internet panel to participate in a survey experiment. Participants were given performance data about 12 fictitious primary care physicians, including a randomized set of narrative feedback from patients. We compared the choice of clinician made by participants who value bedside manner and were exposed to narratives in the experiment, compared to those valuing bedside manner who had not had this exposure. We estimated multivariate logistic regressions to assess whether exposure to patient comments that “disrupt” stereotypes influenced choice of physicians. Participants who saw patient comments and had previously reported caring about bedside manner had a 67% higher odds of choosing a female physician than those participants that did not see a patient comments, controlling for the content of the narratives themselves. When participants were exposed to patient comments that disrupt gendered stereotypes, they had a 40% lower odds of choosing a female physician. Simple exposure to patient narratives that do not clearly disrupt gendered stereotypes increased the likelihood of choosing a female clinician by priming attention to relational aspects of care. However, when the *content* of a sufficient proportion of patient comments runs counter stereotypes, even a minority of narratives is sufficient to disrupt gendered-expectations and alter choices.

## Introduction

Over the past two decades, policy makers around the world have increasingly sought to leverage patient choice among healthcare providers to improve health system performance [1–4]. Although these initiatives promoted choice of hospitals as well as clinicians, they largely focused on encouraging selection among primary care providers [2, 5]. This emerging policy emphasis raises a variety of questions about heretofore understudied aspects of medical consumerism [6, 7].

The U.S. was a front-runner in promoting consumer choice in health care, dating back to the 1970s [8]. This reflected, in part, shifting ideological preferences among policymakers. But it was also a byproduct of a fragmented insurance system that frequently disrupted relationships between clinicians and patients whenever Americans switched jobs or had their employer change health insurers. It has been estimated that roughly 30% of all Americans need to find a new physician each year, including both primary care and specialists, significantly more common than in most other market democracies [9]. Beyond these circumstances, having the option of choosing a physician was also thought to enhance patients’ trust in their physicians by promoting a better match between patient preferences and clinician proclivities [10]. Enhanced trust in turn was shown to be a foundational element of more resilient patient-physician relationships, enhancing the fidelity of communication, compliance with prescribed treatments and the probability that patients would see their clinicians as acting in their best interest of the patient [11].

However, the anticipated virtues of choosing one’s clinician can be compromised when, as is often the case, patients have limited information to guide their choice. Performance metrics are not consistently available for most individual clinicians, since for many clinicians limited sample sizes make it infeasible to calculate reliable outcome-based metrics for their panel of patients that are specific to particular conditions (e.g., asthma, diabetes, hypertension) [12, 13]. The measurement challenges become particularly daunting for primary care clinicians, since their heterogeneous mix of patients (many of whom have neither acute nor chronic conditions) make common condition-specific metrics irrelevant for most of their patients [14]. Furthermore, patients may view the reliability and trustworthiness of external sources of information with varied degrees of skepticism, especially those that offer sketchy documentation about their methods, provenance, or reporting practices [6, 15]. These multiple shortcomings have made it difficult to induce consumers to use or rely upon websites that present comparative performance information about clinicians [7, 16].

When consumers have limited access to trusted measures of quality, they are likely to substitute inferences about quality based on observable physician “attributes”. In other words, they will rely on some form of stereotyping to guide their choices. Past research on consumer selection of clinicians suggests that a number of factors observable through on-line directories may come into play, including the provider’s age, race, gender (typically a binary classification), and place of training [17–20]. How closely these attribute-driven choices mirror more fully informed choice will depend on how much these stereotypes match actual clinician practices and the quality of care they provide [21]. But however close this is, one might still be concerned that stereotype-infused choices risk reifying forms of bias that could be pernicious in other ways.

The challenges of promoting informed selection of clinicians have been evident for the better part of a quarter century [19]. But within the past decade, around the world, a new source of web-based information has become more widespread: patients’ narrative accounts of their interactions with particular clinicians [22–24]. By 2015, narrative comments had become the source of information about clinician quality most frequently consulted by American consumers [22, 25].

Past research documents that consumers find narrative accounts useful [26]; exposure to narratives has been shown to influence which clinicians are selected [19, 27]. But none of this previous research offers clues about how the growing availability of patient narratives might alter the impact of stereotypes in selecting a doctor.

In this paper, we report on an experimental study that offers some unique insights into the circumstances that foster stereotyping in medical consumerism and the potential for patient narratives to buffer these impacts. More specifically, we derive from the extant literature on the psychology of stereotyping some hypotheses that help us examine whether and how the common presumption that female doctors will be more attentive to empathic relationships with their patients shapes consumer choices of primary care clinicians in the absence of metrics that directly measure empathy [19, 28, 29]. In this experimental context, we can then test (through randomized differential exposure to patient narratives) the extent to which patient comments that run consistent or counter to the stereotypes about clinicians’ “bedside manner” have the capacity to alter the impact of stereotypes on consumer choices made when shopping for a new physician.

These gender stereotypes will not apply to all clinicians. Nor will they be equally salient to all patients selecting a new provider. It is only for those patients who value aspects of clinician practice that are not readily observable, and whose understanding of gender stereotypes suggests that knowing this attribute about a clinician will help to predict their practices, that one would expect gender to influence choice of a new doctor [30]. Studying these influences in an experimental context allows us to take into account these complex interactions, while controlling for a variety of other factors that are known to influence how people select a clinician [31].

These comments are randomly assigned through the experimental design to particular clinicians (and thus, to particular clinician genders). Therefore, their introduction could expose any given consumer to narrative content that either reinforces stereotypes or cause consumers to question their reliability [32]. Participants were randomly assigned to different groups (also known as experimental arms); each group/arm was exposed to patient narratives presented in a different format. We make inferences about the impact of stereotyping and the mediating effects of patient narratives based on (a) consumers’ initial expressed preferences (before seeing the website) prioritizing certain aspects of clinician quality, (b) changes in consumer preferences after exposure to the website, (c) the differential exposure across experimental arms to consumer feedback relevant to these preferences, and (d) consumers’ choices among the clinicians available to them.

In the final sections of the paper, we explore some of the policy implications of this interplay between specific forms of evidence about practitioner performance and consumer stereotyping of clinicians. Through stereotyping, individuals make inferences about the expected quality of particular clinicians. Whether this leads to more or less effective sorting and matching of patients with clinicians depends on both the predictive accuracy of the stereotype and consumers’ ability to downplay stereotyping (or otherwise adjust their choices) in the presence of conflicting evidence. Whether reducing the impact of stereotypes improves or worsens patient-clinician matching is therefore a complicated question. We will explore these complications and consider extrapolations to other forms of potential matching that have relevance to contemporary medical consumers and health policy, including racial concordance between patients and clinicians, concerns related to age bias in clinical decision-making, and other related phenomena.

## Background and conceptual framework

“Doctor-shopping” has been studied since the 1960’s. Before comparative information about clinicians was widely available, patients routinely based their choices on word-of-mouth referrals from friends, family, and other physicians [33, 34]. Although sources of information have changed over time, the patterns of what patients’ value when selecting a physician has remained relatively stable: a combination of technical abilities and socio-emotional aspects of the patient-physician relationship [25, 35, 36]. Patients vary in the relative importance they assign to technical skills compared to interpersonal qualities such as “bedside manner” [37–39].

Previous research also suggests that most consumers strike a different balance between technical skills and relational capabilities when selecting a specialist, compared to a primary care clinician. Preferences for specialists more heavily favor technical aspects of quality, whereas choices among primary care providers give greater emphasis to interpersonal ability [21, 40, 41]. The greater importance of interpersonal aspects for choices of primary care clinicians makes sense, since relationships with primary care providers are more likely to be sustained over time and more likely to involve discussions of potentially stigmatized or embarrassing behaviors, enhancing the importance of interpersonal abilities. By contrast, interactions with specialists are more frequently one-off encounters or otherwise short-term interactions [42].

Although metrics of technical quality are only unevenly available for clinicians, metrics assessing empathic interactions (a.k.a. “bedside manner”) are almost entirely absent [36]. Consequently, information about the aspects of clinician performance that many people value the most in primary care settings is largely unavailable. Some consumers can fall back on the anecdotal accounts conveyed word-of-mouth, but this will offer little guidance for those with limited social networks or who have recently moved to places where they know few people [9]. Nor can patients always rely on referrals from other clinicians when selecting a new primary care provider, as they often do when selecting a new specialist [43, 44]. Taken together, these considerations suggest that stereotypes play their most substantial role when consumers are selecting a new primary care provider.

### Potential impact of patient narratives on stereotype-infused choices of clinicians

Social psychologists have long recognized that stereotyping is an inescapable result of the cognitive burdens created by a complex social world [45]. Stereotypes are typically shaped by mass media messaging and other social interactions, as well by individual exposures to members of the stereotyped group [46]. Although stereotypes can sometimes embody negative or demeaning characterizations, our analyses here focus on the decision-making processes related to stereotyping in general, not to the content of any prejudiced thoughts and negative attitudes toward any subset of clinicians [46, 47].

Stereotypes related to gender have been documented for a wide range of social relationships, extending beyond clinical settings and the helping professions. Broadly, these stereotypes emphasize a more “compassionate” representation for women, a more “action-oriented” representation for men [48]. The extent to which these stereotypes shape choice behavior will often depend on how frequently or powerfully they are “activated” by input that makes them feel relevant to the decision at hand [49]. Exposure to comments that describe interactions within clinical settings may, in these ways, either reinforce or erode the perceived relevance to choice of clinicians or alter the perceived validity of the stereotype itself.

Upon first consideration, growing public exposure to patient narratives about interactions with clinicians might be expected to reduce the impact of gendered stereotypes on choices among clinicians. In the absence of information about clinicians’ empathy, stereotypes should lead patients who care about empathy to prefer female providers, all else equal. But patient narratives are rich in content about empathy and other relational aspects of healthcare, conveyed in 30–40% of all reported experiences [25, 36, 42]. By providing concrete information about relationships between patients and clinicians, this evidence ought to disrupt simplistic dualities of gendered stereotyping. We will refer to this as the “naïve learning” hypothesis regarding narratives and stereotyping–not to convey skepticism about the prediction, but to note that this reflects a simple model of learning and updating of prior assumptions related to gender.

**Hypothesis #1: Naïve Learning: Consumers who have patient narratives available for judging clinicians will be less influenced by stereotypes in their choice of clinicians**.

However, a second, countervailing alternative, could also come into play. Narratives convey information about relational aspects of care that would not otherwise be available. But narrative content is not always the easiest information to “process” cognitively–in particular, to identify which clinicians in a given choice set might be the *most* empathic or otherwise most capable of nurturing relationships with a patient [28, 50]. Exposure to patient narratives (many of which talk about empathic relationships with clinicians) might thus increase the salience of relational aspects of care, without providing consumers with enhanced ability to choose based on these aspects. Under these circumstances, the narratives would likely *activate* the stereotype of compassionate female doctors, without conveying evidence that might disrupt the scope or impact of the stereotyping.

**Hypothesis #2**: **Activated Stereotypes: Consumers who have patient narratives available for judging clinicians will display greater influence of stereotypes on choices among clinicians. Stereotype activation will have the opposite influence from naïve learning; which of these two processes will be more influential over choice cannot be predicted *a priori***.

Our review of past research on stereotyped reasoning presented above also suggests a different way in which the introduction of patient narratives could alter choices. To the extent that consumers display motivated reasoning–that is, are predisposed to “defend” their prior beliefs in the face of conflicting evidence–a modest number of exceptions embodied in patient comments may prove insufficient to disrupt established stereotypes, leaving the initial impact of stereotyped reasoning largely unchanged. This suggests that there will be threshold effects–infrequent disruptions of stereotypes leave prior beliefs intact, but frequent disruptions may overturn those prior conceptions.

**Hypothesis #3**: **Motivated Reasoning: Consumers who have patient narratives available for judging clinicians will display no differences in the influence of stereotypes on choices among clinicians, unless the available evidence *strongly* contradicts the stereotypes**.

In short, the literature on stereotyped reasoning suggests that the growing availability of patient narratives could, depending on the underlying causal pathways shaping decision-making, yield a range of potential changes consumer behavior. Some would reduce the impact of stereotypes, others increase them. Later in the paper, we will return to these different pathways to consider how policy might more generally leverage the growing availability of patient narratives to influence the impact of stereotyping on medical consumerism. We next consider a particular test of these impacts on gender stereotyping in choices of primary care physicians.

## Methods

### Study participants

The participants involved in this study were selected from the Growth for Knowledge (GfK) probability-based-Internet Knowledge panel, which is representative of the US population in terms of sociodemographic characteristics, Internet usage, and health status [51]. The Knowledge Panel from which respondents are drawn is composed entirely of panelists from the United States. Following the conventions of this on-line panel, a random sample of 2025 panelists were invited to participate in the study, which was described to them as, “The purpose of the study is to learn how people choose a doctor as their regular source of medical care and advice. The study is being conducted by researchers at several major universities and research organizations including Yale and UCLA.” Participants were recruited to this study between the Summer of 2014 and the Summer of 2015.

All members of the panel are offered a modest non-monetary incentive to participate in surveys and complete 1–4 studies each month. 52% of the individuals invited to participate in this study accepted their invitation [56]. All participants underwent an informed consent procedure approved by IRB’s at Yale University and the RAND Corporation. Electronic consent was obtained from all participants for inclusion in this study. We did not have access to information that could identify individual participants at any time point during or after the study.

### Experimental design and procedure

After agreeing to join the study, participants completed a pre-experiment survey. They answered questions about their real-life health experiences, including their experiences making choices among clinicians. This information was collected to ensure that randomization had successfully allocated participants with comparable levels of prior experience to each arm of the experiment. Respondents were also asked to describe their preferences regarding clinician characteristics. All told, participants were asked about the importance of a dozen clinician attributes. Among these was one attribute involving clinicians’ relational capabilities: if a physician being “warm, caring and a good listener” was important to them, an attribute we anticipate being most closely linked to gender stereotypes. (A matching question was included in the post-choice survey to assess whether exposure to the website had altered preferences).

**Fig 1:**
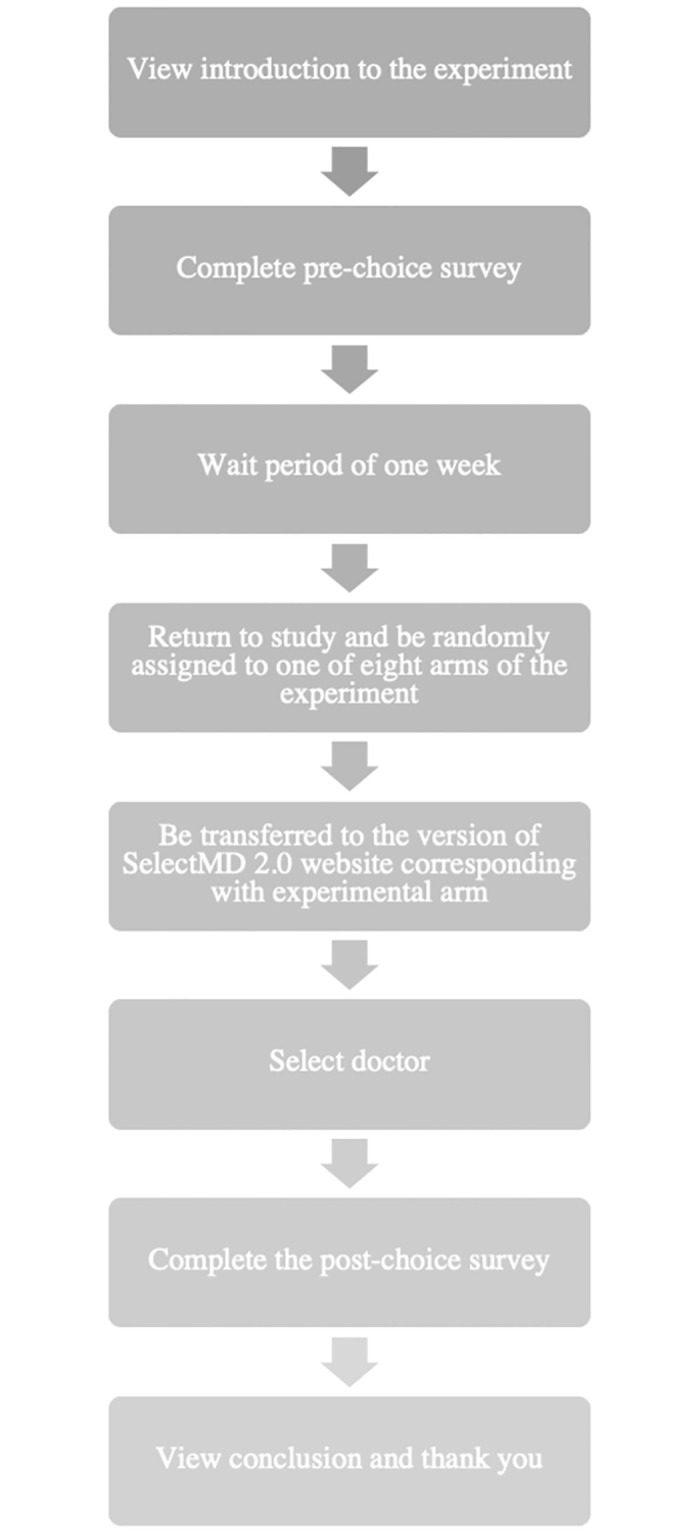
Flow diagram of the SelectMD 2.0 provider choice experiment.

Roughly a week after completing this survey, respondents were invited to return to the study and participated in the choice experiment. (The week-long delay was designed to ensure that responses on the pre-choice survey did not induce a consistency bias in respondent’s choice of clinician or expressed rationale for the choice.) The various stages of the experiment are outlined in Fig 1. A total of 85% of those who completed the pre-choice survey returned to participate in the choice experiment. Choosing among clinicians called for logging onto a “SelectMD” website of our own design, on which participants were given information about 12 fabricated primary care physicians and asked to select their preferred clinician among these options. Clinicians were listed in random order on the website. Participants expressed few concerns with the functionality of the website; 90% reported that the site was “somewhat easy or very easy to use” [35]. Participants generally spent between 5 and 7 minutes on the website before selecting a preferred clinician.

**Fig 2:**
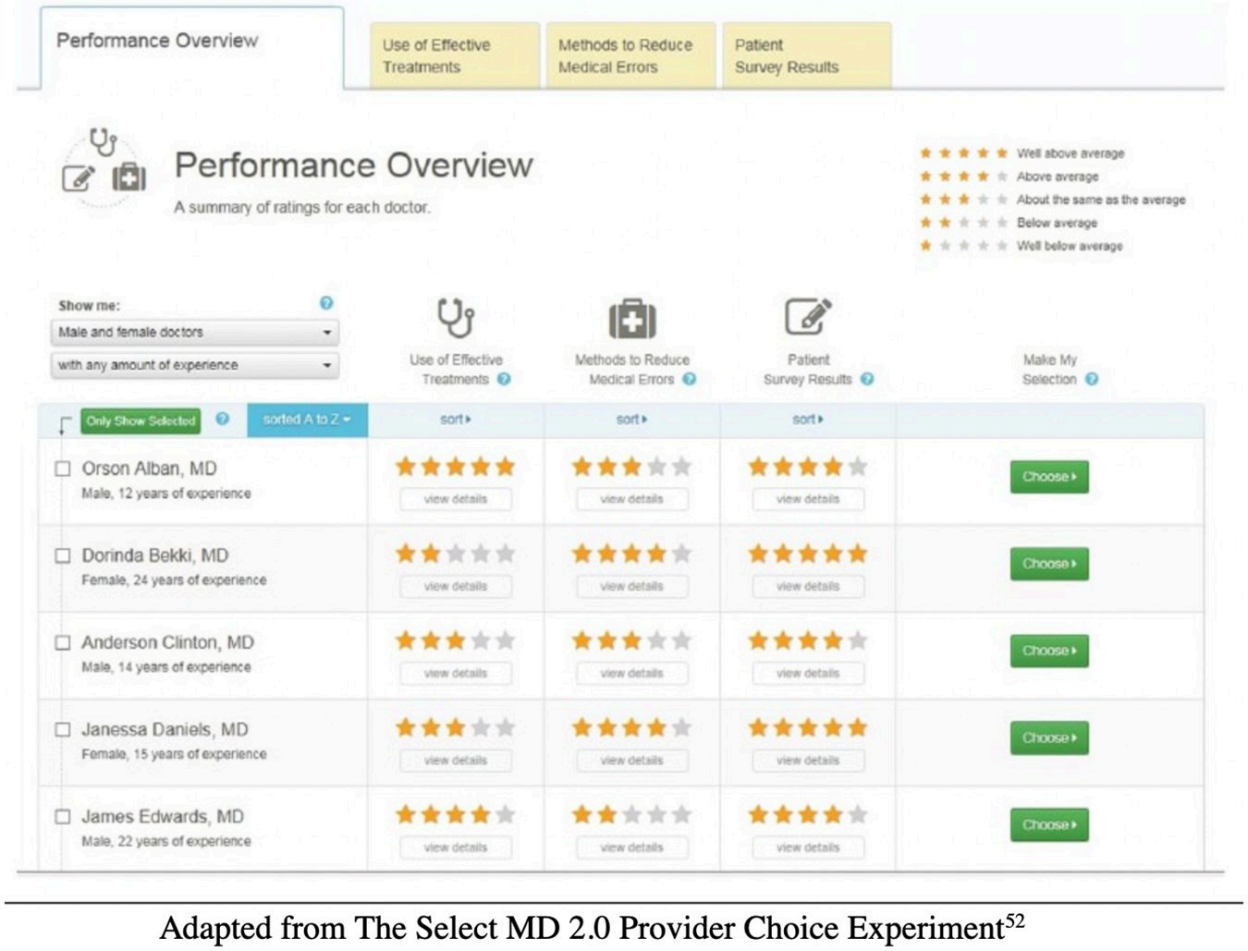
Performance rating displays seen by participants on the “Select MD” website.

All participants were exposed to an identical distribution of quantitative performance measures across the 12 fabricated clinicians. These measures included star ratings (1–5 stars, with five stars representing the highest-rated clinicians) associated with three aspects of quality: (a) a rating for effective communication drawn from patient experience surveys, (b) a rating for the clinician’s ability to deliver care consistent with norms of good practice, drawn from clinical records and (c) a rating for how comprehensively the clinician’s practice used safety-enhancing protocols (Fig 2). Consistent with data observed in real-world clinician choice websites, the quality and safety ratings were loosely correlated (r = 0.15), the communication ratings uncorrelated with the other two metrics for quality.

The star ratings were balanced in several ways with respect to physician gender (Table 1). Aggregating across the three categories, the top-rated clinicians based on star-metrics (all with 12 stars in total) included two female and two male clinicians. Male and female clinicians were equally likely to be top-rated (five star) and bottom-rated (one star) across the three quantified ratings. Female doctors had an average performance rating of 9.1 while male doctors had an average performance rating of 8.8 (Table 1). Because our prior research on consumer choice revealed that a number of consumers used a satisficing strategy across multiple dimensions of performance (e.g., avoid selecting a clinician who was below average on any rated dimension), we also balanced by gender clinicians who were at or above average (3+ stars) across all three performance categories. Doing so also allowed us to avoid respondents who may have made hasty choices in choosing a physician.

**Table 1:** Fixed attributes for fabricated clinicians in Select MD 2.0.

Identifiers			Star Ratings for:		
First Name	Last Name	Gender	Clinical Effectiveness	Safety Practices	Communication Effectiveness
Jannette	Sharp	Female	4 stars	4 stars	1 star
Nikolas	Oliver	Male	5 stars	5 stars	2 stars
Elmira	Mathews	Female	2 stars	2 stars	2 stars
Bonnie	Malandra	Female	1 star	2 stars	2 stars
Tony	Lambert	Male	2 stars	1 star	3 stars
Dorinda	Bekki	Female	2 stars	4 stars	5 stars
Orson	Alban	Male	5 stars	3 stars	4 stars
Janessa	Daniels	Female	3 stars	4 stars	5 stars
Candice	Gaines	Female	4 stars	5 stars	3 stars
Anderson	Clinton	Male	3 stars	3 stars	4 stars
James	Edwards	Male	4 stars	2 stars	4 stars
Tyson	Porter	Male	1 star	1 star	1 star

**Fig 3:**
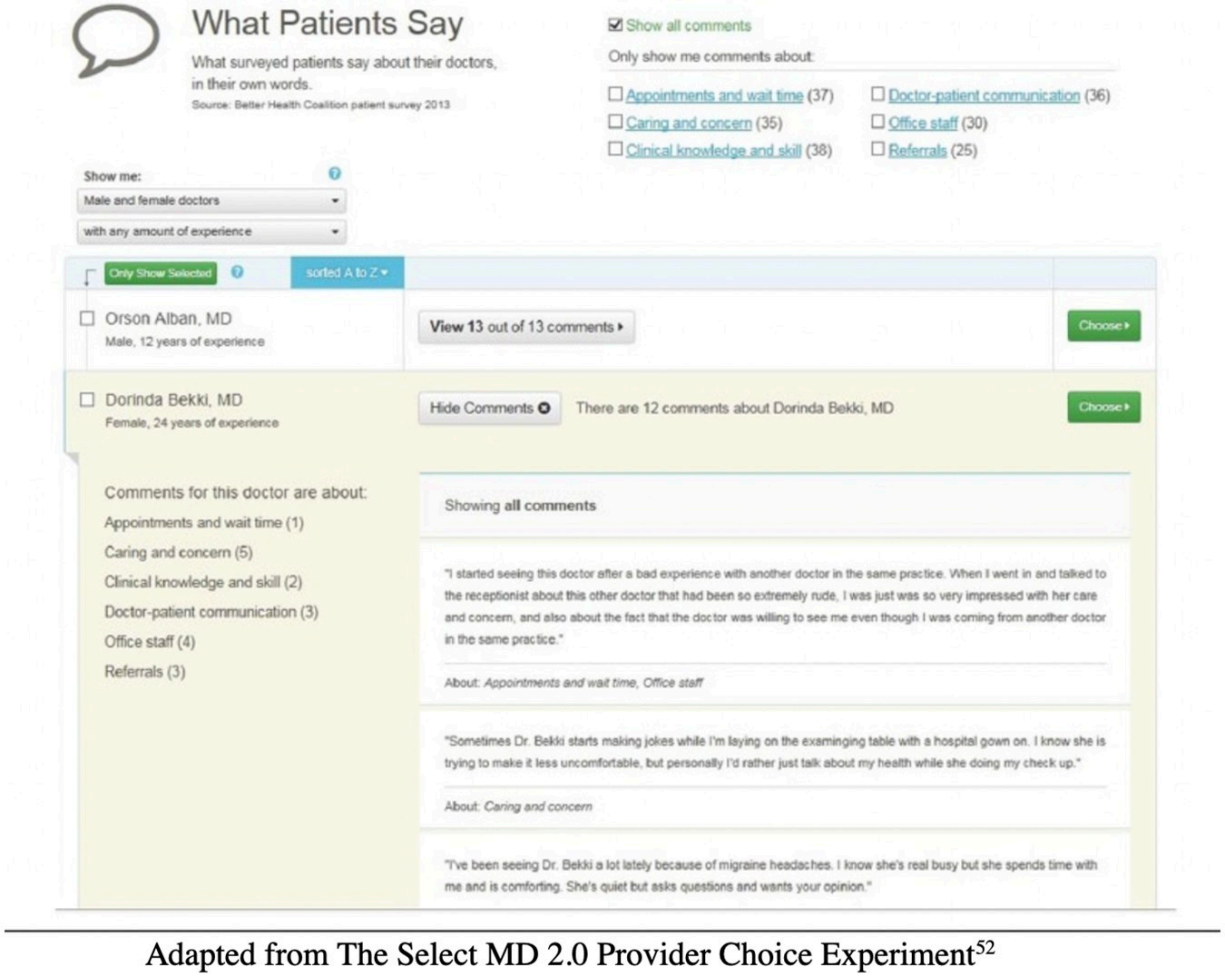
Comments displays seen by participants on the “Select MD” website.

By contrast to the fixed configuration of star ratings, exposure to specific patient comments was randomized. Comments were drawn from a pool of 142 real-world comments harvested from internet sites, grouped into four different categories of clinician performance, one of which was empathy/emotional rapport [35]. When the narratives were randomized and assigned to each clinician, pronouns were adjusted in the text of each relevant comment to match the gender of the clinician to which the narrative had been assigned. Any given website user was exposed to an average of 12 comments, drawn from four categories of clinician performance (Fig 3). The research team assigned a valence (ranging from negative to positive on a five-point scale) to each comment; these assigned valences were validated against scores assigned by members of the public to the same comment set. Five members of the research team, drawn from different disciplines (psychology, management, and economics) independently rated the valence for each comment and the modal score was then assigned to that comment. (This valence was never reported to website users, but had to be inferred by them from the content of the narrative comment.) Here again, consistent with observed patterns from real-world websites, the valence of the aggregated patient comments for each clinician was loosely correlated (r = 0.15) with the star rating derived from the patient experience survey scores.

Because the specific comments (and associated valences) were randomized for each participant, any one person visiting the SelectMD website could encounter patient comments related to empathy/emotional rapport that were fully consistent with gender stereotypes, fully inconsistent with those stereotypes, or a mix of consistent and inconsistent narratives. And since the number of narratives in the empathy/emotional rapport domain was also randomly varying, the strength of the “signal” conveyed by the narratives (however consistent or inconsistent) also varied across participants, subjecting stereotypes to different levels of challenge from the content of the narratives [50].

In keeping with a broader experimental design, participants were randomized into one of seven arms [52]. In all but one of these arms, participants were given the ability to read patients’ comments about each clinician (Table 2). The arms varied in terms of the formatting used to present the patient comments and the capacity that website users had to sort among the comments. The names and genders of physicians were visible to participants in all arms of the experiment (Figs 2 and 3).

**Table 2:** Summary of the seven experimental arms of the SelectMD 2.0 experiment.

Arm	Drill-Down Scores	Roll-up Scores	Patient Comments	Special Features
1. Standardized measures only (Control group)	✓	✓		None
2. Standardized measures with comments (experimental baseline group)	✓	✓	✓	None
3. Roll-up scores only, plus comments		✓	✓	None
4. Drill-down scores only, plus comments	✓		✓	None
5. Amazon Model	✓	✓	✓	When viewing patient comments, respondents could see how commentators rated the positive or negative nature of their own comments and the distribution of positive-to-negative comments (much like how comments are displayed on the Amazon Website).
6. Tagged Comments	✓	✓	✓	When viewing patient comments, respondents could use a list of keywords to view only comments that addressed specific topics.
7. Navigator	✓	✓	✓	Content and display were the same as Arm 6 but respondents could talk to a patient navigator by phone while viewing the SelectMD site and deciding which doctor to choose.

Note: Adapted from The Select MD 2.0 Provider Choice Experiment [52].

After participants selected a clinician on the website, they completed a post-choice survey asking about their experiences on their website, their decision-making process, and satisfaction with the choices given to them. This included questions about whether (a) respondents had noticed that there were patient comments on the website (not all had) and (b) how effectively they were able to extract information from these comments to compare across clinicians (not all were). As part of this sequence of questions, they were asked about which attributes of physicians mattered to them most while making their selection. This question was worded identically to the question about preferences included on the pre-choice survey.

### Key analytic variables and constructs

The primary dependent variable in this study was the choice of clinician. A binary outcome variable was constructed using the gender of the chosen clinician, to identify when a female clinician physician was chosen. A value of one denotes a female physician; a value of zero denotes a male physician (no value-laden inferences are intended by this coding).

Past research on patients’ assessing clinicians based on their gender suggest that stereotypes will alter choices only for those patients who value the aspects of clinician practice that are related to gendered expectations–in this case, those who place a particularly strong value on “bedside manner”. Because this was assessed in the pre-choice survey (a week prior to participating in the experiment), these preferences were not influenced by the content of the website. 23 percent of respondents placed bedside manner among the top three (out of the dozen possibilities) attributes that they valued in a clinician.

Our initial analyses of the impact of narratives compare the choices made by those who value bedside manner and were exposed to narratives in the experiment, compared to those valuing bedside manner without this exposure. But “exposure” is a relatively complex categorization. For purposes of our analyses presented below, the control group (that is, those not exposed) was constructed as an amalgamation of three subsets of participants. The first group includes participants randomized to the Arm 1 of the experiment, that is, those individuals who did not have any patient narratives incorporated into their version of the SelectMD website (N = 174). The second group includes participants from Arms 2 through 7 (all of whom narratives on their version of the website) who reported in the post-choice survey that they did not actually access the comments on the website (N = 246). The third group includes participants who reported having seen the comments but felt unable to identify from the narratives which doctor was best in the choice set using the narratives were also added to the augmented control group (N = 124). Consequently, our baseline tests of the impact of patient narratives on stereotyped decision-making among those who both saw and felt able to interpret the content of the narrative accounts. From a total sample of 1,052 participants, 564 participants were in the treatment group while 488 were in the control group.

Constructing the control and treatment groups in this manner provides a meaningful test for a particular form of narrative impact–influencing choices among those who felt they could derive meaning from the patient comments. Because there are other plausible ways to define narrative exposure (each having different interpretations regarding narrative impact), we will explore some alternative definitions of treatment and control groups later in the paper.

Our earlier review of the literature on motivated reasoning suggests that the potential impact of patient narratives will also depend on the informational content of the narratives–in particular, the extent to which they convey experiences that run consistent with or counter to the gendered stereotypes. Because comments are randomized for each participant, some will have received a mix of narratives related to bedside manner that reinforce stereotypes, others a mix of comments that include a few narratives that are inconsistent with gender stereotypes (and therefore weakly disruptive of the stereotypes), yet others a mix of comments that disproportionately represent male clinicians as caring/empathic and female clinicians as emotionally aloof.

Participants were exposed to anywhere from zero to twelve stereotype disrupters and were on average exposed to 4 stereotype disrupters comments. For our baseline analyses, we first measured comments that defied the gender stereotype (henceforth referred to as the “stereotype disrupter” variable) as a *continuous* variable: more specifically, as the proportion of all available comments that conveyed experiences that ran counter to the gender stereotype. As we define the gender stereotype as female doctors being more caring and having better bedside manner skills than male doctors, thus the stereotype disrupter is when male doctors are described as particularly warm and caring or female doctors as not so. The coefficient conveys the marginal impact of exposure to an additional disruptive comment on the probability of selecting a female physician from the choice set of twelve primary care clinicians.

In the final regression specification, we represented exposure to stereotype disrupter comments based on the proportion of comments (these were clustered to increase the stability of the estimates) that disrupted gender stereotypes. This allows us to identify whether there are non-linear effects in the ways that narrative comments disrupt stereotypes and to identify the threshold at which disruptive comments become consequential for choosing a physician.

### Statistical analysis

To assess whether simple exposure to comments (with their random distribution of valence across genders) altered the propensity to select clinicians who were female, we initially estimate a multivariate logistical regression model. The outcome of interest is the interaction term between being exposed to patient comments (Treatment Group) and caring about bedside manner (Bedside Manner). This tests for the difference in the effect of exposure to patient comments on choice of a female physician, for participants who value bedside manner, relative to those who do not have that preference OR who were not exposed to the narratives.

Next, using a second multivariate logistical regression model, we tested the effect of stereotype disrupters on choice of physician. The outcome of interest is stereotype disrupter defined as a continuous variable. Thus, we are able to test for the difference in the effect of being exposed to stereotype disrupter patient comments on choice of female physician.

## Results

The analyses included 1,052 participants. Compared to the general US public (as per the 2019 American Community Survey [53]), our sample was slightly older, more White, and less likely to be in in excellent health (Table 3). In addition, participants did not differ across the treatment and control group in terms of any health or demographic characteristic, with the exception of the proportion of individuals in the Age Group from 30–44 (S1 Table in S1 File). Since there were no differences in most of these characteristics between treatment and control group, they were not included as covariates in the models presented, which the exception of Age, which was included as a covariate. Overall, 51% of participants chose a female physician.

**Table 3:** The experimental sample.

	Experimental Sample		US Population
	Mean	SD	
**Demographic Characteristics (%)**			
*Age Group*			
18–34	20.53	0.40	23.1
35–49	20.82	0.41	18.8
50–64	34.69	0.47	19.1
65+	23.95	0.43	16.9
Female	47.33	0.49	51.3
*Race/Ethnicity*			
White	77.94	0.41	75.3
Black	7.22	0.26	14.0
Other Race	9.51	0.29	14.2
Hispanic	5.32	0.22	18.0
*Education Level*			
High School or less	34.31	0.47	39.0
Some College	26.90	0.44	29.1
College Graduate	38.78	0.49	32.1
**Health Status**			
*Self-reported health*			
Excellent	11.12	0.31	17.4^a^
Very Good	40.87	0.49	31.5 ^a^
Good	35.83	0.48	32.3 ^a^
Fair/Poor	10.46	0.30	18.8 ^a^
**N**	1052		

^a^From the KFF analysis of the Centers for Disease Control and Prevention (CDC)’s 2013–2019 Behavioral Risk Factor Surveillance System (BRFSS) [1, 34].

Because the model focuses on the choices of those who gave priority to bedside manner, it behooves us to consider how much this priority was affected by exposure to the website. Therefore, we compare preferences for bedside manner before and after the experiment. Roughly a quarter of the sample shifted their reported priority for this attribute. But an equal number of respondents increased the priority given to bedside manner as did those who decreased their priority. Because our primary interest is in assessing the impact of patient comments on the use of stereotypes for a given set of preferences, our baseline models use the pre-choice preference ranking. (Later in the paper we replicate these models using the post-choice preferences, allowing us to assess whether exposure to patient comments also alters preference rankings).

### Effect of patient comments on choice of physician

To assess the initial effect of exposure patient comments on choice of physician, the first model estimates the probability of choosing a female physician comparing participants who saw patient comments and reported caring about Bedside Manner to those who did not see any patient comments or who did not care about bedside manner. Those who saw comments *and* cared about the relational aspects of care had a 67% higher odds of choosing a female physician than participants who did not see a patient comments (p = 0.083). The results do not change when controlling for age (Table 4).

**Table 4:** Exposure to patient comments, bedside manner, and choice of physician.

Explanatory Variables	Odds of choosing Female Physician	Odds of choosing Female Physician
Exposure to Comments • Care about Bedside Manner	1.676* (0.49)	1.676* (0.49)
Care about Bedside Manner	0.917 (0.205)	0.917 (0.205)
Exposure to Comments	0.871 (0.123)	0.866 (0.122)
Age	-	0.998 (0.263)
N	1052	1052

Coefficients represented as Odds Ratios. Robust standard errors in parentheses.

* p <0.1,

** p<0.05,

*** p<0.01

### Effect of stereotype disruptive comments on choice of physician

**Fig 4:**
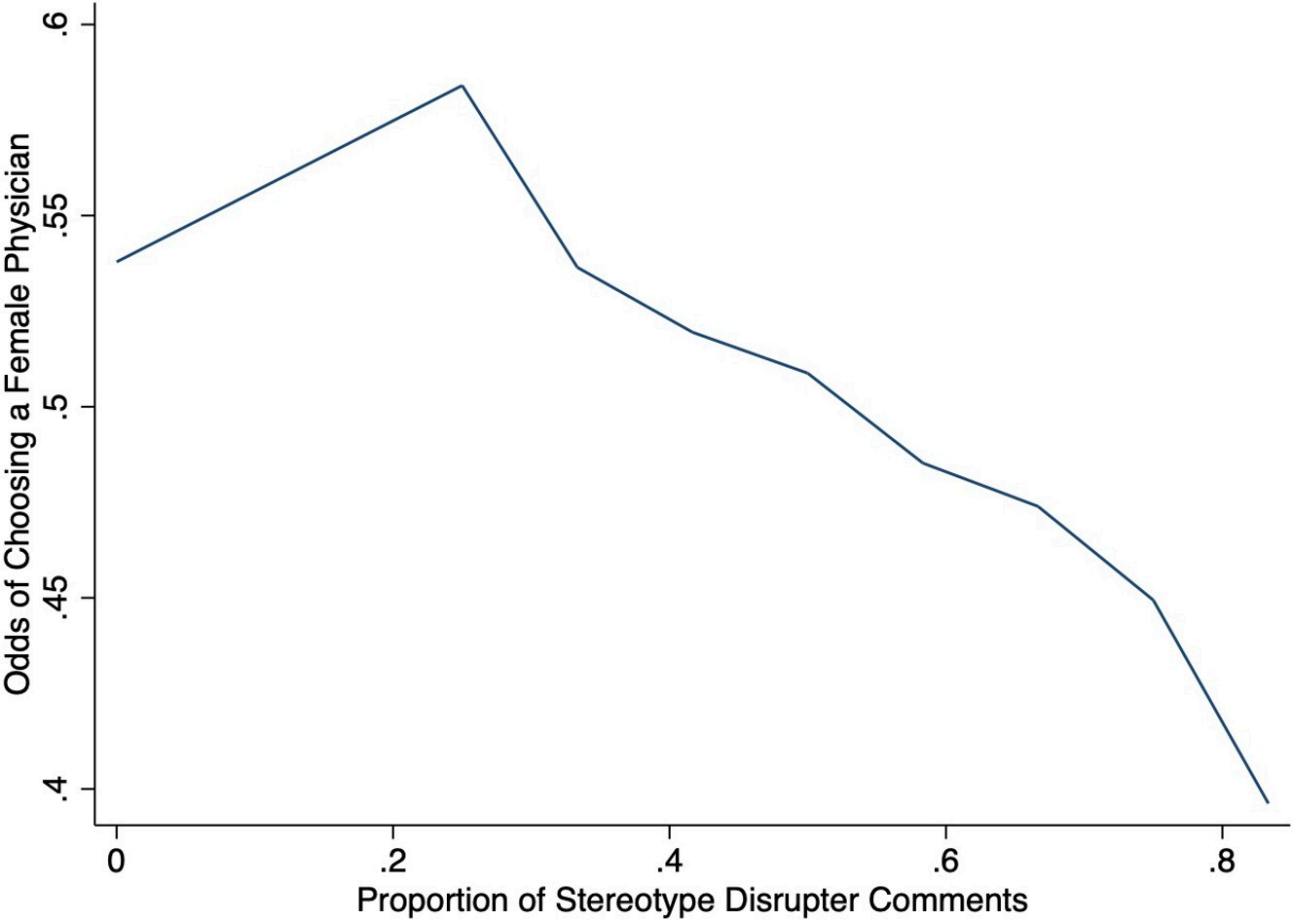
Odds of choosing a female physician by the proportion of stereotype disrupter comments.

Measuring stereotype disrupters as a continuous variable shows that for every additional stereotype disrupting comment, there is a 6.8% lower odds (p = 0.016) of choosing a female physician for participants exposed to comments. Here again, the results do not change when controlling for age (Table 5, left column). Fig 4 graphically represents these findings. It illustrated the negative relationship between the odds of choosing a female physician and the number of stereotype disrupter comments on the website. But it also suggests that the relationship may incorporate a threshold effect.

**Table 5:** Exposure to “stereotype disrupter” comments by varied definitions.

	Exposure to “stereotype disrupters” (continuous)	Exposure to “stereotype disrupters” (paired clusters^a^)
Explanatory Variables	Odds of choosing Female Physician	Odds of choosing Female Physician
25–34% Stereotype Disrupters	-	1.227 (0.537)
40–50% Stereotype Disrupters	-	0.605** (0.144)
55–65% Stereotype Disrupters	-	0.542** (0.125)
Over 70% Stereotype Disrupters	-	0.700 (0.269)
Stereotype Disrupter	0.932* (0.027)	-
Care about Bedside Manner	1.244 (0.184)	1.253 (0.186)
Exposure to Comments	1.383* (0.268)	1.442* (0.303)
Age	0.998 (0.004)	0.998 (0.003)
N	1052	1052

Coefficients represented as Odds Ratios. Robust standard errors in parentheses.

* p <0.1,

** p<0.05,

*** p<0.01

^a^ Clustered pair for 1–2 stereotype disrupter comments was omitted due small sample

To test for these threshold effects, we re-estimated the model with the proportion of disruptive comments clustered into five strata. The results suggest that when exposed to comments wherein at least 40% of patient narratives are disrupting gendered stereotypes, participants were significantly less likely (odds-ratio 0.605, p = 0.035) to select a female physician than those participants who saw fewer or no disruptive comments (Table 5, right column). These findings suggest there is in fact a threshold for disrupting stereotypes. But that threshold is relatively low. Even when consumers are exposed to *less* than a majority of comments that run counter to stereotypes, there may still be a sufficient accumulation of disruptive comments to measurably reduce the odds of choosing based on gendered stereotyping.

### Sensitivity analyses

To ensure that our estimates of the impact of stereotypes are not unduly sensitive to model specification, we tested a variety of alternative specifications which are presented in the S1 File. First, because past research suggests that exposure to patient narratives can cause some consumers to become distracted from quantifiable (star-rated) performance rankings [27], we wanted to ensure that the apparent reduction in stereotyped selection was not a byproduct of consumers who had become overloaded with the complexity of the choices we faced. To do this, we limited the sample to those whose final selection avoided doctors who scored below average on any of the three star-rated dimensions. Using this restricted sample, we then re-estimated the regressions. None of these alternative specifications significantly altered the results presented above (S2 Table in S1 File).

In our baseline specifications presented above, we treated as equivalent disruptive comments conveying positive bedside manner for male physicians and those that portrayed the bedside manner of female clinicians in negative terms. But there is no reason to presume that these forms of disruption will have equal impact. In subsequent model specifications, we assessed stereotype disrupters, stratified by physician gender, allowing for stereotype disrupters to have potentially different effects for male and female physicians (S3 Table in S1 File). Here again, we observe no significant differences from the findings reported for the baseline models.

Since we know from our findings reported above that exposure to the website led a quarter of our participants to alter their pre-experiment preference of valuing bedside manner, we wanted to assess whether this might alter the apparent impact of comments on stereotyping. This involved re-estimating all the models, but in this case the group for which stereotypes were deemed to be salient was identified based on their expressed preference for bedside manner preference *post*-experiment. Here again, this revised specification did not significantly alter any of our findings (S4-S6 Tables in S1 File). Our results were also robust to non-linear effects of stereotype disrupters (S7 Table in S1 File).

## Discussion

The findings from this study provide insights into some ways in which stereotyping shapes consumers’ choices among clinicians. First and foremost, stereotypes clearly seem to affect the selection of a primary care physicians. The impact of stereotyping on choice becomes more pronounced when consumers encounter reports of other patients’ experiences. In this context, for those participants who reported valuing a “warm and caring” physician, being exposed to patient comments increased their chances of choosing a female physician, holding constant the content of these comments. Second, the impact of stereotyping can be altered by the content of other patients’ narratives. For those participants that value bedside manner, being exposed to a sufficient proportion of comments that run counter to stereotypes decreased the chance of choosing a female clinician. This aligns with the Activated Stereotype Hypothesis, since exposure to patient narratives led to a greater influence of stereotypes in choosing a physician. Disruption of stereotypes is evident even if there is only moderate exposure to narratives that run counter to the stereotype. Although these findings need to be interpreted in the context of certain methodological limitations discussed below, they hold some potentially important implications for both the understanding of stereotyping in medical settings as well as for clarifying how health policies might address (and potentially mitigate) that impact.

### Better understanding how stereotyping affects medical consumerism

Simple exposure to patient narratives increased the likelihood of choosing a female clinician, consistent with our Activated Stereotype Hypothesis. This pattern also comports with findings from the literature on priming effects related to other complex health choices, [54] as well as those from studies of the impact of stereotyping on voter selection among political candidates [54]. But it may initially seem counterintuitive, when the content of so many of the narratives presented on the SelectMD website are inconsistent with gendered stereotyping of clinicians.

The explanation rests in the complicated calculus of choosing a physician and the often-challenging task of translating narrative accounts into choice preferences. Patients value many skills and attributes in clinicians. Of the dozen attributes that respondents were asked about on the pre-experiment survey, the modal respondent gave high priority to eight of the attributes. That is a lot of attributes to balance when selecting a preferred provider.

These are precisely the circumstances in which narrative priming can be most consequential. Roughly 40% of real-life narratives about doctors involve what we have labeled here as “bedside manner” [36]. Consumers who casually peruse these comments are reminded that this aspect of medical care matters to the experiences of many other patients. Even if they don’t attend closely enough to the content of the comments to try to discern which doctors perform best in terms of these relational aspects of care, the salience of “bedside manner” rises for their selection of a primary care clinician.

It is clear that a certain proportion of consumers will not probe narratives that closely. 15.7% of the participants in the choice experiment indicated that it was hard for them to discern from the narratives which clinicians were performing best. Those consumers will favor the simpler, star-rated assessments of clinician practices, precisely because those can be readily rank-ordered. If consumers don’t pay attention to the narrative content that disrupts stereotypes, the heightened salience of bedside manner activates those stereotypes and makes gender more influential in choice of a physician. Thus, the subset of individuals who value bedside manner within the 15.7% of participants who did not necessarily use patient narratives to choose their physician, are more likely to rely on physician gender stereotypes to find a physician that they believe can fulfill their bedside manner needs.

However, when consumers *do* pay attention to the content of patient comments and these comments frequently run counter to stereotypes, gendered expectations can be disrupted. That requires that at least a third of comments are contrary to stereotypes. In this context, consumers do not appear to strongly “defend” their preconceived gender stereotypes (as some theories of motivated reasoning might predict), since they appear willing to alter their choices away from stereotyped preconceptions, even if a majority of comments remain consistent with stereotypes.

How might these insights extend to stereotyping based on other observable attributes such as clinicians’ race or age? Clinicians age and race may induce different types of stereotypes than does gender [55, 56]. Whether these stereotypes would be triggered in the absence of narratives will depend on other information that is available on real-world websites (but not presented on our experimental site), including photo images of the clinician or the year in which they graduated from medical school. To whatever extent stereotypes are triggered by real-world websites designed to support clinician choice, it seems plausible that the stereotype dampening effects of narratives would likely extend to these additional clinician attributes, however this should be tested with further research. Patients’ preference for clinicians who seem “similar” to them is associated with their assessed threat of unfair treatment and their hopes that matching will ease communication, comfort, and trust [57]. However, though impaired communication and perceived mistreatment are reported less frequently in racially and ethnically concordant pairings [58, 59], communication failures and perceived discrimination are sufficiently common in these contexts that the stereotypes supporting racial matching are a rather unreliable predictor of high-quality interactions between patients and providers [60, 61].

Might matching be enhanced if consumers had greater access to patients’ narrative accounts of their interactions with providers? For some aspects of care–and some motivations for seeking out racially concordant pairings–patients’ comments could signal otherwise unmeasured sources of communication breakdowns, lack of empathy, or untrustworthy practices, since all these aspects of care are regularly reported in patients’ narratives about their treatment [36]. However, to the extent that provider selection is motivated by concerns about racially-targeted discrimination, comments seem a less reliable remedy. Patients less often report on “unfair” treatment in ways that explicate the sources of discriminatory practices. And when they do so, their own race or ethnicity may be difficult to discern from the comment, making it difficult for consumers to determine which patients are at greatest risk for discrimination.

Conversely, patient narratives may offer a more reliable way to address flawed stereotypes related to age. Many patients are predisposed toward younger physicians [61], presuming that more recently trained clinicians will have better technical, emotional, and explanatory skills than older doctors [62]. Because these broad age-related stereotypes are often inaccurate characterizations for particular clinicians and because these domains of clinician-patient interaction are widely reported-on in patient narratives, there seems to be considerable opportunity for patient comments to erode age-related biases in the same way that they disrupted gendered stereotypes.

### Stereotyping and the opportunities for policy intervention

The implications of these findings suggest that when individuals are given information derived from patient accounts inconsistent with their preconceived notions, they adjust their beliefs using the new “data” presented to them. Whether these lead to better choices, however, depends on our capacity to elicit narratives from a broad cross-section of patients and to do so in ways that encourages narratives that provide a high-fidelity representation of how it feels to get care from a particular clinician. In recent years, the capacity to rigorously elicit patient narratives has improved [63]. But one cannot presume that the comments that are currently available on websites are necessarily either representative or complete, leading to other potential biases in consumer choice.

A potentially more constructive stance for health policy would involve treating the overall supply of patient comments as a form of public good. This characterization is easy to recognize in countries that have a unified health care delivery and financing system, like the National Health Services in the U.K [64]. But it is more difficult to comprehend–and infinitely more difficult to implement—in an American context that remains committed to promoting competition among providers. Over the past decade, there has been a dramatic proliferation of healthcare systems collecting patient narratives about their affiliated clinicians and posting those narratives on-line [6, 12]. However, since these narratives are being deployed, at least in part, for strategic advantage, there are ample incentives for systems to suppress feedback that offers a more negative assessment of their patients’ experiences.

Under these circumstances, there are two potential pathways toward preserving a more reliable and robust reservoir of patient comments that can be made freely accessible to consumers. The first approach would involve creating and enforcing norms of good practice for narrative reporting by each individual healthcare system. A second approach would be to assign government (at state and/or federal levels) the responsibility for eliciting and posting patient narratives [12]. This could involve collecting comments from patients of all ages, covered by all forms of insurance. Or it could be more focused on the substantial portions of the American public who already have their healthcare financed through Medicare or Medicaid. If the latter approach was taken, it would then be straightforward to take comments collected from Medicare and Medicaid beneficiaries and make them freely available to the general public, much as all Americans can already access the Medicare Compare website to review comparative performance data on many types of health care providers and health plans [65].

### Generalizations and the need for additional research

The analyses presented above examined consumers’ choices among primary care clinicians. But the findings may also have implications for the medical specialties. Past research suggests that stereotypes about physician gender lead to patients preferring a female physician for dermatology, pediatrics, obstetrics/gynecology, and family medicine [66]. Likewise, patients prefer male physicians for surgical specialties such as orthopedic surgeons [57]. To the extent that actual clinical behavior fails to match these stereotypes for one or more of these specialties, a shortage of comments addressing relational aspects of care may lead to consistent mismatching of patient preferences and provider practices. The capacity for patient comments to disrupt stereotypes in the short-run thus portends the longer-term potential to alter consumer choices in ways that reshape the ways in which aspiring clinicians (a.k.a. medical students and residents) select into particular specialties.

These study findings need to be interpreted in the context of some methodological limitations. First, this was a hypothetical choice experiment. Thus, participants may engage with the experiment with less consistent or probing attention than they would if the choices had actual consequences for their medical care. This may imply that our results are an underestimate of the effect that learning from narratives can have on stereotyped choices, as participants in real life would be more likely to carefully engage with the website material (and thus process more fully narrative content which is more difficult to interpret than counting stars) if this was a real-life decision to be made with more consequential stakes. Second, the choice set in this experiment was structured to be carefully balanced according to gender in terms of quantitative performance metrics. In a real-world context, where there will be less balance in other performance metrics, the impact of stereotypes may skew access to other desirable clinician attributes.

In addition, stereotyping may also shape how patients interpret their interactions with clinicians themselves, so stereotypes can have a more profound and lasting impact on the physician-patient relationship. As a result, the impact of stereotypes may extend to the interpretation of subsequent patient experiences, in that seeing stereotype reinforcing patient comments may lead to a patient to interpret an interaction with a physician as also stereotype reinforcing, which they themselves will report. Also, the presumption that female physicians will be more caring and warmer than their male counterparts is only one of several gender stereotypes. Future research could help to more carefully parse the multiple ways in which current and potential patients interpret and anticipate outcomes based on clinician gender. In addition, in order to fully assess the impact of gender stereotypes, further analyses are needed, studying other gender stereotypes of clinicians, including the common assumption that male physicians are more technically equipped than female physicians [39, 58, 67, 68].

## Conclusion

Most patients want to select their primary care clinician with minimal effort and attention, especially those who see themselves in relatively good health [9, 35]. These are precisely the circumstance in which stereotyping is likely to have the most pronounced influence on choices. Online patient narratives can provide access to information about aspects of clinician skills and behavior that are not reliably measured by other means, helping consumers to choose based on some evidence, rather than relying entirely on stereotypes. How much this might improve matching of patient preferences and clinicians’ skills will depend on the availability of narratives that reflect a broad range of patient experiences and convey those experiences in reliable ways. That can and should become a focus for policymakers intending to promote consumer choices as a way to improve health system performance.

## Supporting information

S1 File (DOCX)
