# Supplementary material for: How exposure to patient narratives affects stereotyped choices of primary care clinicians
Source: PLoS One. 2023 Dec 7;18(12):e0295243. doi: 10.1371/journal.pone.0295243 (PMC10703228; doi:10.1371/journal.pone.0295243)
Supplement: S1 File — (DOCX) [file pone.0295243.s001.docx]

**How Exposure to Patient Narratives Affects Stereotyped Choices**

**of Primary Care Clinicians**

**Online Supplement**

**Table S1** Baseline Characteristics of the Experimental Sample

**Table S2** Varying definitions of stereotype disruption adjusted for physician rating

**Table S3** Exposure to stereotype disrupters by physician gender

**Table S4** Exposure to patient comments, choice of physician, and bedside manner preference Post-Experiment

**Table S5** Varying thresholds of stereotype disruption using Post-experiment preferences

**Table S6** Exposure to paired clusters of stereotype disrupters using Post-experiment preferences

**Table S7** Non-linear impact of exposure to “stereotype disrupter” comments (continuous)

**Appendices**

| Table S1. The Experimental Sample | | | | | | |
| --- | --- | --- | --- | --- | --- | --- |
|  | Treatment Group | | Control Group | | T-test Difference | |
|  | Mean | SD | Mean | SD | Difference | P-Value |
| Demographic Characteristics (%) |  |  |  |  |  |  |
| *Age Group* |  |  |  |  |  |  |
| 18-34 | 0.23 | 0.42 | 0.18 | 0.39 | -0.04 | 0.09 |
| 35-49 | 0.24 | 0.43 | 0.17 | 0.38 | -0.07 | 0.01 |
| 50-64 | 0.33 | 0.47 | 0.36 | 0.48 | 0.03 | 0.26 |
| 65+ | 0.20 | 0.40 | 0.28 | 0.45 | 0.08 | <0.01 |
| Female | 0.47 | 0.50 | 0.48 | 0.50 | 0.01 | 0.71 |
| *Race/Ethnicity* |  |  |  |  |  |  |
| White | 0.78 | 0.42 | 0.78 | 0.41 | 0.00 | 0.93 |
| Black | 0.07 | 0.25 | 0.08 | 0.26 | 0.01 | 0.68 |
| Other Race | 0.05 | 0.22 | 0.06 | 0.23 | 0.01 | 0.58 |
| Hispanic | 0.10 | 0.30 | 0.09 | 0.28 | -0.02 | 0.36 |
| *Education Level* |  |  |  |  |  |  |
| High School or less | 0.35 | 0.48 | 0.34 | 0.47 | -0.01 | 0.75 |
| Some College | 0.27 | 0.44 | 0.27 | 0.45 | 0.01 | 0.81 |
| College Graduate | 0.39 | 0.49 | 0.39 | 0.49 | 0.00 | 0.93 |
| Health Status |  |  |  |  |  |  |
| *Self-reported health* |  |  |  |  |  |  |
| Excellent | 0.12 | 0.32 | 0.10 | 0.30 | -0.02 | 0.40 |
| Very Good | 0.42 | 0.49 | 0.39 | 0.49 | -0.03 | 0.35 |
| Good | 0.35 | 0.48 | 0.37 | 0.48 | 0.03 | 0.36 |
| Fair/Poor | 0.09 | 0.29 | 0.12 | 0.32 | 0.03 | 0.16 |
| Chosen their own Health Care Provider in the past | 0.94 | 0.23 | 0.94 | 0.24 | -0.00 | 0.86 |
| N | 564 | | 488 | | 1052 | |

| Table S2. Varying definitions of stereotype disruption adjusted for physician rating | | |
| --- | --- | --- |
|  | Exposure to “stereotype disrupter” (continuous) | Exposure to *any* “stereotype disrupter” |
|  | Odds of choosing Female Physician | Odds of choosing Female Physician |
| Stereotype Disrupter | 0.918** (0.032) | 0.566** (0.129) |
| Care about Bedside Manner | 1.485**  (0.267) | 1.487**  (0.267) |
| Exposure to Comments | 1.620** (0.373) | 1.627** (0.305) |
| N | 720 | 720 |
| Coefficients represented as Odds Ratios. Robust standard errors in parentheses.  * p <0.1, ** p<0.05, *** p<0.01 | | |

| Table S3. Exposure to stereotype disrupters by physician gender | | |
| --- | --- | --- |
|  | Exposure to Male Physician stereotype disrupters (continuous) | Exposure to Female Physician stereotype disrupters (continuous) |
|  | Odds of choosing Female Physician | Odds of choosing Female Physician |
| Stereotype Disrupter | 0.862  (0.141) | 1.152 (0.242) |
| Care about Bedside Manner | 1.224  (0.180) | 1.223  (0.180) |
| Exposure to Comments | 0.980  (0.122) | 0.977 (0.121) |
| N | 1052 | 1052 |

| Table S4. Exposure to patient comments, choice of physician, and bedside manner preference Post-experiment | | |
| --- | --- | --- |
|  | Bedside Manner preference  Pre-experiment  *(Original Specification)* | Bedside Manner preference Post-experiment |
|  | Odds of choosing Female Physician | Odds of choosing Female Physician |
| Exposure to Comments • Care about Bedside Manner | 1.676*  (0.49) | 1.557*  (0.384) |
| Care about Bedside Manner | 0.917  (0.205) | 1.188  (0.205) |
| Exposure to Comments | 0.871  (0.123) | 0.671  (0.292) |
| N | 1052 | 1052 |
| Coefficients represented as Odds Ratios. Robust standard errors in parentheses.  * p <0.1, ** p<0.05, *** p<0.01 | | |

| Table S5. Exposure to stereotype disruptors (continuous) using Post-experiment preferences | | |
| --- | --- | --- |
|  | *Original Specification* | Post-experiment preferences |
|  | Odds of choosing Female Physician | Odds of choosing  Female Physician |
| Stereotype Disrupter | 0.932*  (0.027) | 0.933** (0.027) |
| Care about Bedside Manner | 1.244  (0.184) | 1.197  (0.180) |
| Exposure to Comments | 1.383*  (0.268) | 1.384* (0.267) |
| N | 1052 | 1052 |
| Coefficients represented as Odds Ratios. Robust standard errors in parentheses.  * p <0.1, ** p<0.05, *** p<0.01 | | |

| Table S6. Exposure to paired clusters of stereotype disrupters using Post-experiment preferences ^a^ | | |
| --- | --- | --- |
|  | Exposure to “stereotype disrupters” (Pre-experiment) | Exposure to “stereotype disrupters” (Post-experiment) |
|  | Odds of choosing Female Physician | Odds of choosing Female Physician |
| 25-34%  Stereotype Disrupters | 1.216  (0.532) | 1.230  (0.537) |
| 40-50%  Stereotype Disrupters | 0.605**  (0.144) | 0.613**  (0.146) |
| 55-65%  Stereotype Disrupters | 0.541**  (0.125) | 0.551**  (0.126) |
| Over 70%  Stereotype Disrupters | 0.696  (0.268) | 0.689  (0.265) |
| Care about Bedside Manner | 1.251  (0.185) | 1.195  (0.180) |
| Exposure to Comments | 1.454*  (0.304) | 1.444*  (0.303) |
| N | 1052 | 1052 |
| Coefficients represented as Odds Ratios. Robust standard errors in parentheses.  * p <0.1, ** p<0.05, *** p<0.01  ^a^ Cluster of <25% stereotype disrupter comments omitted due to small subsample | | |

| Table S7. Exposure to “stereotype disrupter” comments (continuous with quadratic term) | | |
| --- | --- | --- |
| Explanatory Variables | Odds of choosing Female Physician (original) | Odds of choosing Female Physician |
| Stereotype Disrupter | 0.932** (0.027) | 0.836*  (0.077) |
| Stereotype Disrupter ^2 | - | 1.013  (0.011) |
| Care about Bedside Manner | 1.243  (0.184) | 1.253  (0.186) |
| Exposure to Comments | 1.392* (0.269) | 1.527**  (0.317) |
| N | 1052 | 1052 |
| Coefficients represented as Odds Ratios. Robust standard errors in parentheses.  * p <0.1, ** p<0.05, *** p<0.01 | | |
